# Supplementary material for: Patterns of Intron Gain and Loss in Fungi
Source: PLoS Biol. 2004 Nov 30;2(12):e422. doi: 10.1371/journal.pbio.0020422 (PMC532390; doi:10.1371/journal.pbio.0020422)
Supplement: Table S1 — Also available at http://genes.mit.edu/NielsenEtAl/. (4.3 MB ZIP). [file pbio.0020422.st001.zip › NielsenEtAl/html/1445.html]

AN0158.1.NCU04443.1.MG06586.1.FG05690.1


```
 CLUSTAL W (1.82) Multiple Sequence Alignments - Introns Inserted


Sequence 1: AN0158.1	332 aa
Sequence 2: NCU04443.1	336 aa
Sequence 3: FG05690.1	329 aa
Sequence 4: MG06586.1	332 aa
Alignment Length: 348 aa
Number Identitical Residues: 91 aa
Alignment Score (without introns) 5408


MG06586.1 	-----MATMRAVAFDKTGDSSVLSLREDMPKPTASASQILVKVEWHGINFIDT~YMRTGL
NCU04443.1	MAAEIPKTMSGVVIEKPGGVDVLKYKTDLPVPEIKEGELLVKNEYIGVNFIDT2YFRTGL
FG05690.1 	-MAPVPSTMKAVQINKNGGVEVLEHN-DVPVPKVGEGQILVRNQIAGLNFIDT~YFRSGL
AN0158.1  	--MPPLSTMKAIVTEKTGGPEVLEVRTHHPVPTASEGQLLVKNQIIGINYIDT~YFRTGL
          	       ** .:  :* *. .**. . . * *    .::**: :  *:*:*** *:*:**

MG06586.1 	YQFATPPPHVSGTEGGGIVEAVGAEVPADYGLEVGDRVAFMKSP-SAAEYAAVNPNLCFK
NCU04443.1	YPSASGYPLITGKEASGTVVSS----KHPR-FSPGDRVAYLGDA-AYAEYTAVSASRCIG
FG05690.1 	YKAPQ-FPLTLGREAAGTIVDIH---SSVKGFENGSRVVFMGTVGAYAQYSVVNASDAVK
AN0158.1  	YPSSK--PEILGREAVGEVVAVGPG-ANPYNFQIGDRVAWLSTG-SYAEYTAVPQAFAVK
          	*  .   *   * *. * :    .      .:. *.**.::    : *:*:.*    .. 

MG06586.1 	-LPEGVSTKDGAAFLLQGLTAWTLVRDAHEVKP------GEVVLVQAAAGGTGGLVVQMC
NCU04443.1	PLPERISSETAAAALLQGLTALTLVREAAGIEQKLGISEGPWALVHAAAGGTGSLLVQIL
FG05690.1 	-IPDDVPTEQAVAAYLQGLTAWTFIREAGQVKE------GQWVLVHAASGGVGTLLVQLL
AN0158.1  	-VPEGISNEDVMGSFLSGLTVLTLAKETYPVQR------GDWVLLHAAAGGAGFLMTQVL
          	 :*: :..:   .  *.***. *: :::  ::       *  .*::**:**.* *:.*: 

MG06586.1 	KHLGATVIGTVSTDAKAKLAREHGCDHVVVYTRD---DVLKAVNEITHDLGCHAVFSGVG
NCU04443.1	SIMGAKVIATAGGQEKCEQAHHHGAHWTIDNKSE---DVVERVKEITNGRGVDVIFDGVG
FG05690.1 	RTVGAKIIGTASTDEKLELAKKNGAEWVISSHDD----VVAKVKEITGGHGADVIFDGVG
AN0158.1  	KSIGAIVIGTAGGPEKVELVKSLGADYVIDYRSEEGKDWVKKVKEITNGRGVDVVYDSVG
          	  :** :*.*..   * : .:  *.. .:    :.... :  *:*** . * ..::..**

MG06586.1 	KATFEQDLAATRRKGSFITFGNA~SGAVEPFSLF~KLTARNVRLMRPTLFNFVATREEWD
NCU04443.1	KATFDADLEMVARKGTVISFGNA~SGKVEPLDIL~RLGAKNIKVMRPVLFGYIVTPEEWE
FG05690.1 	KVTFDADLELIAPKGTLISFGNA0SGAVDPVSLF~RLTPKNVRLMRPVVNGYVAQRADLE
AN0158.1  	KDTWEGSLEAVKRKGMIVWFGNA~SGPVPPLPLA2KLSPKCVKVARPQLFGYIQAREEFE
          	* *:: .*     ** .: **** ** * *. :  :* .: ::: ** : .::    : :

MG06586.1 	ARCRELFDLLAKGVVKTHIGGVYDLSEVAKAQDDLTGQKTVGKLLVKVAKD
NCU04443.1	RWTTELFELIKTEKVKIKVHEVYPLQEVARAHTDLEGRKTTGKLLLKL---
FG05690.1 	KYTSELFDLIKSKKLELTIHKVYPLADVAKAHEDIESRKTIGKLLIKVD--
AN0158.1  	FYVNELFNMLKSGNLKVKIHKVYPLEQVAEAHSDLEGRKTTGKLLLKA---
          	    ***::: .  ::  :  ** * :**.*: *: .:** ****:*
```
